# Aortic root dilation in bicuspid aortic valve disease: impact on dissection risk and evaluation of guideline-based surgical thresholds — a retrospective cohort study

**DOI:** 10.1097/JS9.0000000000002657

**Published:** 2025-06-05

**Authors:** Haoyu Gao, Xiang Liu, Yuqiang Wang, Zhenghua Xiao, Bin Shao, Jun Shi, Xiaogang Sun, Chang Shu, Yingqiang Guo

**Affiliations:** a Department of Cardiovascular Surgery, West China Hospital, Sichuan University, Chengdu, China; b Cardiovascular Surgery Research Laboratory, West China Hospital, Sichuan University, Chengdu, China; c Information Technology Center, West China Hospital, Sichuan University, Chengdu, China; d Department of Cardiovascular Surgery, State Key Laboratory of Cardiovascular Disease, Fuwai Hospital, National Center for Cardiovascular Diseases, Chinese Academy of Medical Sciences and Peking Union Medical College, Beijing, China; e Center of Vascular Surgery, State Key Laboratory of Cardiovascular Disease, Fuwai Hospital, National Center for Cardiovascular Diseases, Chinese Academy of Medical Sciences and Peking Union Medical College, Beijing, China

**Keywords:** acute aortic dissection, aortic root dilation, bicuspid aortic valve aortopathy, guideline model

## Abstract

**Introduction:**

To evaluate the impact of aortic root dilation in patients with bicuspid aortic valve (BAV) and to assess guideline thresholds and real-world surgical practices to determine the optimal intervention timing for Stanford type A aortic dissection (AAD).

**Methods:**

From 2008 to 2023, 1582 BAV patients with an aortic diameter ≥40 mm were retrospectively analyzed and categorized into root-dilation (aortic root ≥40 mm) and non-root-dilation groups. AAD risk associated with root dilation was assessed using Firth’s penalized Cox regression, inverse probability of treatment weighting-adjusted Cox regression, and Fine–Gray competing risk models. Inflection points were identified via restricted cubic spline (RCS) analysis and validated through receiver operating characteristic and decision curve analysis. Guideline-based thresholds from the 2014 ESC, 2022 ACC/AHA, and 2024 ESC guidelines were evaluated, and real-world surgical practices were analyzed for comparison.

**Results:**

The mean age was 55.8 ± 12.9 years; the median follow-up was 27 months [IQR 19–49]. Root-dilation patients exhibited faster growth of the aortic root (0.86 ± 1.82 vs. 0.77 ± 1.90 mm/year) and ascending aorta (0.93 ± 2.12 vs. 0.79 ± 2.04 mm/year; both *P* < 0.001). Root dilation conferred over a two-fold increased AAD risk. RCS identified 45 mm (root) and 50 mm (ascending aorta) as key inflection points. The 2024 ESC model had the highest predictive accuracy (AUC = 0.731), with further improvement using revised thresholds (AUC = 0.752). Surgery was performed in 65.3% of patients with root diameters of 45–50 mm, compared to 47.9% with ascending aortic diameters in the same range. Surgery at a 45 mm root diameter was associated with an 88% reduction in AAD risk.

**Conclusions:**

Root dilation in BAV patients is associated with faster aortic growth and a higher AAD risk, supporting earlier surgical intervention. The 2024 ESC guidelines performed best, but further refinement of thresholds may be needed.

## Introduction

Bicuspid aortic valve (BAV) is the most common congenital heart defect, with a prevalence of approximately 0.5–2% in the general population^[1-3]^. Besides aortic valve dysfunction, one of the most concerning conditions is the heterogeneity of aortopathy risk. Previous studies have shown that patients with BAV are at an increased risk of aortopathy, including a significantly higher risk of Stanford type A aortic dissection (AAD) compared to the general population^[4,5]^. Understanding the characteristics and progression of these aortic abnormalities is crucial for improving the management and outcomes of BAV patients.

**HIGHLIGHTS**

- The study included 1582 patients with BAV-related aortic pathology. Aortic root dilation is a high-risk phenotype in BAV aortopathy, with ≥45 mm for the root and ≥50 mm for the ascending aorta identified as critical inflection points for AAD risk.
- The 2024 ESC model demonstrated the highest predictive accuracy for AAD, and real-world surgical practice, which often involves intervention at root diameters of 45–50 mm, further supports the rationale for earlier surgical treatment of BAV-related aortopathy.
- Lowering the surgical thresholds to 45 mm for the aortic root and 50 mm for the ascending aorta was associated with improved AAD prediction, suggesting that earlier intervention may be beneficial in selected BAV patients with root dilation.

To improve risk stratification for BAV-related aortic disease, an international consensus statement has classified aortopathy into three phenotypes based on the location of dilation: ascending aorta type, root type, and extended type[6]. Among these phenotypes, the ascending aorta type is the most common but is generally considered the most stable. In contrast, the root type, which accounts for approximately 15–20% of cases, is regarded as a more severe aortic pathology, often associated with a faster growth rate^[7,8]^. Reflecting the heightened risk, the latest European Society of Cardiology (ESC) Guidelines and European Association for Cardio-Thoracic Surgery and The Society of Thoracic Surgeons (EACTS/STS) Guidelines have adjusted the surgical threshold for root-type BAV-related aortopathy to ≥50 mm and ascending type to 52 mm, with a Class I level of recommendation, emphasizing the need for earlier intervention in these patients^[9,10]^.

The optimal timing for surgical intervention in ascending aortic disease remains a topic of ongoing debate. Recent guidelines have lowered the surgical threshold to 53 mm from traditional recommendations. However, a more recent study suggests that intervention should occur when the ascending aorta reaches 50 mm, as the risk of adverse aortic events rises sharply at this point. The variability in disease progression among BAV patients complicates decision-making, especially when considering different aortic phenotypes. This is particularly true for patients with root-type aortic dilation. Current guidelines may still be conservative, despite emerging evidence supporting earlier intervention in certain high-risk phenotypes ^[4,8,11]^. This gap highlights the need for further research to refine surgical thresholds, especially for different aortic types in BAV-related aortopathy, where the optimal timing for intervention remains uncertain.

This study hypothesizes that each phenotype exhibits a distinct progression pattern, with varying growth rates and differing risks of AAD, especially in root-type, necessitating further refinement of surgical indications for BAV-related aortic pathologies. By conducting a retrospective analysis, we aim to determine the natural history and risk of AAD in BAV patients with different ascending aortic phenotypes, thereby identifying the optimal timing for surgical intervention in each phenotype. Additionally, we will construct a risk model to evaluate the robustness of recent aortic disease guidelines concerning the surgical indications for BAV-related aortic disease.

## Method

### Patient population

This study included all patients diagnosed with BAV through transthoracic echocardiography (TTE). Each echocardiogram was independently reviewed by two experienced echocardiographers, followed by a consensus review to confirm the diagnosis^[12,13]^. From January 2008 to June 2023, a total of 7069 patients were diagnosed with BAV via TTE. Among them, 2980 patients presented with an aortic diameter (either ascending aorta or aortic root) greater than 40 mm at baseline evaluation and had at least one echocardiographic follow-up. After excluding patients aged <18 years, those diagnosed with Marfan syndrome, and those with incomplete echocardiographic or clinical data, 1582 patients were ultimately included in the study. The study was reported in line with the STROCSS criteria^[14,15]^.

### Data collection and end points

Aortic root and ascending aorta diameters were determined by measuring the maximum internal dimension at the sinuses of Valsalva and the mid-ascending aorta, respectively. Aortic dilation was defined when the maximum aortic diameter was ≥40 mm. If the aortic root diameter was ≥40 mm, the condition was classified as root-dilation group. If the aortic root was <40 mm but the ascending aorta measured ≥40 mm, the case was considered non-root dilation group. In this study, we used the baseline aortic diameter for all analyses. The aortic growth rate was defined as: Aortic growth rate = Aortic diameter at last TTE − Baseline  aortic diameter,  Aortic stenosis (AS) and aortic regurgitation (AR) were diagnosed using both quantitative and qualitative analysis via TTE. This study primarily focused on moderate or greater valvular disease for further analysis. Additionally, TTE data were used to quantitatively collect information on end-systolic volume (ESV), end-diastolic volume (EDV), ejection fraction (EF), and left ventricular posterior wall thickness (LVPW). Demographic data and clinical characteristics were gathered from the hospital’s electronic medical records system.

The primary endpoint of this study was AAD, with aortic surgery treated as a competing risk event. This study was a retrospective observational study, with the observation starting at the patient’s first TTE diagnosis of BAV at our institution. The observation ended at the patient’s last TTE examination, with an interval of more than 1 year.

### Estimation of aortic growth rate

To assess the growth rates of the ascending aorta and aortic root between groups, we first used a linear model to identify variables associated with the growth rate. Subsequently, a generalized additive model (GAM) was applied to account for potential nonlinear relationships.

### Risk modeling for AAD

To evaluate the impact of aortic root dilation, variable selection was conducted using univariate Cox regression and LASSO regression. Multivariate modeling was then performed using three approaches: Firth’s penalized Cox regression to address potential small-sample bias, inverse probability of treatment weighting (IPTW) combined with Cox regression to adjust for baseline confounding, and a Fine–Gray subdistribution hazards model to account for aortic surgery as a competing risk for AAD. A restricted cubic spline (RCS) analysis was used within the Cox model to flexibly model the association between baseline aortic size and AAD risk. Inflection points for AAD risk were identified by calculating the first derivative of the spline. To further evaluate the predictive power of inflection points for AAD, we constructed receiver operating characteristic (ROC) curves for different follow-up aortic diameters, increasing in 5 mm increments (45, 50, and 55 mm). Decision curve analysis (DCA) was performed to assess whether the identified inflection points fell within the clinically optimal thresholds for surgical intervention.

### Evaluation of BAV-related aortopathy guideline models

This study primarily evaluated the recommendations for surgical timing in BAV-associated ascending aortopathy proposed by three major international guidelines, as well as a hypothetical model developed in this study. The guidelines included the 2014 ESC Guidelines, the 2022 American College of Cardiology/American Heart Association (ACC/AHA) Guidelines, and the newly released 2024 ESC Guidelines and 2024 EACTS/STS Guidelines^[9,10,16,17]^. To simplify the analysis, we extracted key variables from each guideline, including aortic diameter, phenotypic classification, and dilation rate. By developing risk models, we compared the predictive performance of these guidelines’ recommendations for AAD risk. The dependent variable for all models was AAD, while the independent variables are shown in Figure 5A. Additionally, we compared the predictive performance of the 2014 ESC, 2022 ACC/AHA, and 2024 ESC guidelines, as well as a hypothetical model developed in this study, by constructing ROC curves for each model to assess their ability to predict AAD risk.

### Fine–Gray modeling of AAD risk and surgical timing

To investigate the impact of real-world surgical timing on the risk of AAD, we applied the Fine–Gray competing risks model under two distinct modeling strategies. One strategy incorporated aortic surgery as a competing event, reflecting actual clinical practice in which surgical intervention may preclude the occurrence of AAD. The other strategy excluded competing events, thereby estimating the counterfactual risk of AAD progression in the absence of surgical intervention.

### Statistical analysis

Continuous variables were expressed as mean ± standard deviation or median [interquartile range (IQR)]. Statistical tests were conducted using either the *t*-test or the Wilcoxon rank-sum test, as appropriate. Categorical variables were presented as percentages, and comparisons were made using the Chi-square test or Fisher’s exact test. Patients were classified into two groups based on aortic phenotype: root-dilation group and non-root dilation group. Statistical tests were performed between the root-dilation and non-root dilation groups, with a *P* value < 0.05 considered statistically significant. Variables with a *P* value < 0.1 in univariate analyses were included in subsequent analyses. All analyses were conducted using R statistical software (version 4.2.1, R Foundation for Statistical Computing).

## Results

### Study population and baseline characteristics

A total of 1582 patients with aortic pathology were included in this study, comprising 518 patients in the aortic root dilatation group and 1064 in the non-root dilatation group. The mean age across all patients was 55.79 ± 12.92 years. Patients in the root dilatation group were significantly younger compared to those in the non-root dilatation group (54.28 ± 13.48 vs. 56.52 ± 12.57 years; *P* value = 0.002). The root-dilation group had a significantly higher proportion of male patients compared to the non-root dilation group (91.51% vs. 59.21%; *P* value < 0.001). The mean body mass index (BMI) was 23.28 ± 3.63 kg/m^2^, and the mean body surface area (BSA) was 1.66 ± 0.18 m^2^, with no significant differences observed in BMI or BSA between the two groups. Comorbid conditions included hypertension (*n* = 251), chronic obstructive pulmonary disease (*n* = 33), coronary artery disease (*n* = 108), hyperlipidemia (*n* = 31), chronic kidney disease (*n* = 32), type 2 diabetes mellitus (*n* = 95), prior stroke (*n* = 24), history of smoking (*n* = 410), and atrial fibrillation (*n* = 74). The prevalence of these comorbidities did not differ significantly between the groups (Table 1).

**Table 1:** Baseline characteristics

Variable	Overall (*n* = 1582)	Root dilation (*n* = 518)	Non-root dilation (*n* = 1064)	*P* value
Age, y	55.79 ± 12.92	54.28 ± 13.48	56.52 ± 12.57	0.002
Male, *n* (%)	1104 (69.79)	474 (91.51)	630 (59.21)	0.000
BMI, kg/m^2^	23.28 ± 3.63	23.19 ± 3.66	23.32 ± 3.61	0.435
BSA, m^2^	1.66 ± 0.18	1.66 ± 0.18	1.66 ± 0.18	0.990
Hypertension, *n* (%)	251 (15.87)	87 (16.80)	164 (15.41)	0.527
COPD, *n* (%)	33 (2.09)	10 (1.93)	23 (2.16)	0.909
CAD, *n* (%)	108 (6.83)	39 (7.53)	69 (6.48)	0.505
Hyperlipidemia, *n* (%)	31 (1.96)	9 (1.74)	22 (2.07)	0.802
CKD, *n* (%)	32 (2.02)	10 (1.93)	22 (2.07)	1.000
Smoking, *n* (%)	410 (25.92)	145 (27.99)	265 (24.91)	0.210
Type II diabetes, *n* (%)	95 (6.01)	32 (6.18)	63 (5.92)	0.929
History of stroke, *n* (%)	24 (1.52)	4 (0.77)	20 (1.88)	0.124
AF, *n* (%)	74 (4.68)	24 (4.63)	50 (4.70)	1.000

AF, atrial fibrillation; BMI, Body mass index; BSA, body surface area; CAD, coronary artery disease; CKD, chronic kidney disease; COPD, chronic obstructive pulmonary disease; SD, standard deviation. Values are *n* (%), mean (SD).

### Echocardiographic findings

All participants had complete echocardiographic data. The mean ascending aorta diameter was 44.8 ± 5.54 mm. The average ascending aorta diameter was significantly smaller in the root dilatation group compared to the non-root dilatation group (43.48 ± 7.28 vs. 45.44 ± 4.32 mm; *P* value < 0.001). The mean aortic root diameter was 36.39 ± 6.35 mm, with the root dilatation group demonstrating a larger mean diameter (43.68 ± 3.98 mm) compared to the non-root dilatation group (32.85 ± 3.72 mm; *P* value < 0.001). A total of 798 patients had moderate or greater AR, with a significantly higher prevalence observed in the root-dilation group compared to the non-root dilation group (67.58% vs. 42.41%; *P* value < 0.001). AS and other indicators of cardiac function, including ESV, EDV, EF, and LVPW, did not differ significantly between the root-dilation and non-root dilation groups (Table 2).

**Table 2:** Echocardiographic results

Variable	Overall (*n* = 1582)	Root dilation (*n* = 518)	Non-root dilation (*n* = 1064)	*P* value
Ascending aortic diameter, mm	44.8 ± 5.54	43.48 ± 7.28	45.44 ± 4.32	0.000
Aortic root diameter, mm	36.39 ± 6.35	43.68 ± 3.98	32.85 ± 3.72	0.000
Ascending aortic index, mm/m^2^	26.46 ± 5.00	30.69 ± 3.93	24.41 ± 4.09	0.000
Aortic root index, mm/m^2^	21.29 ± 3.93	22.36 ± 4.36	20.77 ± 3.58	0.000
ESV, ml	58.98 ± 48.05	57.84 ± 44.8	59.55 ± 49.6	0.565
EDV, ml	142.52 ± 71.33	142.16 ± 68.15	142.7 ± 72.89	0.183
EF,	61.84 ± 11.54	62.09 ± 11.11	61.72 ± 11.74	0.954
LVPW, mm	10.35 ± 2.11	10.22 ± 1.91	10.42 ± 2.2	0.165
AR (moderate or above), *n* (%)	798 (23.45)	350 (67.58)	448 (42.11)	0.000
AS (moderate or above), *n* (%)	674 (42.60)	216 (41.70)	458 (43.05)	0.650

AS, Aortic stenosis; AR, aortic regurgitation; EDV, end-diastolic volume; EF, ejection fraction; ESV, end-systolic volume; LVPW, left ventricular posterior wall thickness; SD, standard deviation. Values are *n* (%), mean (SD).

### Aortic growth rate

**Figure 1.**
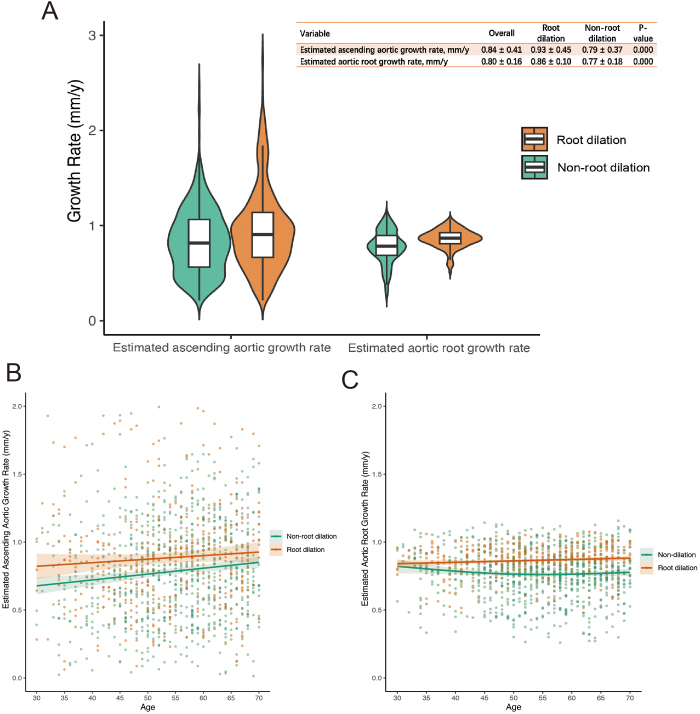
Growth rates of the ascending aorta and aortic root in patients with root and non-root dilation. (A) Violin plots illustrating the estimated ascending aortic growth rate and aortic root growth rate in patients with root dilation (orange) and non-root dilation (green). The growth rates are expressed in millimeters per year (mm/year). The accompanying table summarizes the mean ± SD and *P* values of the *T*-test for comparisons between the two groups. (B) Scatter plot with regression lines showing the relationship between age and the estimated ascending aortic growth rate in patients with root dilation (orange) and non-root dilation (green). The regression curves are fitted using GAMs, with shaded regions representing the 95% confidence interval (CI). (C) Scatter plot with regression lines depicting the association between age and the estimated aortic root growth rate for the two patient groups. The regression curves are fitted using GAM, with shaded regions representing the 95% CI. CI, confidence interval; SD, standard deviation.

At the end of follow-up, the mean ascending aortic diameter was 47.2 ± 7.31 mm, and the mean aortic root diameter was 38.69 ± 8.06 mm. The growth rate of the ascending aorta was significantly higher in the root dilatation group compared to the non-root dilatation group (0.93 ± 2.12 vs. 0.79 ± 2.04 cm/year; *P* value < 0.001). Similarly, the growth rate of the aortic root was also significantly higher in the root dilatation group (0.86 ± 1.82 vs. 0.77 ± 1.90 cm/year; *P* value < 0.001) (Table 3). Variables associated with the growth rates of the ascending aorta and aortic root were initially identified using linear models and subsequently incorporated into GAM models (Supplementary Table 1, http://links.lww.com/JS9/E294). The estimated aortic growth rates adjusted using the GAM model are shown in Figure 1. In the root-dilation group, the estimated growth rates for both the ascending aorta and aortic root remained significantly higher than those in the non-root dilation group. This difference persisted across all ages, with the root-dilation group showing consistently greater growth rates at each age point.

**Table 3:** Follow-up results

Variable	Overall (*n* = 1582)	Root dilation (*n* = 518)	Non-root dilation (*n* = 1064)	*P* value
Ascending aortic diameter, mm	47.2 ± 7.31	46.64 ± 9.24	47.47 ± 6.15	0.039
Aortic root diameter, mm	38.69 ± 8.06	45.76 ± 5.85	35.25 ± 6.61	0.000
Ascending aortic growth rate, mm/year	0.84 ± 2.07	0.93 ± 2.12	0.79 ± 2.04	0.000
Aortic root growth rate, mm/year	0.80 ± 1.88	0.86 ± 1.82	0.77 ± 1.90	0.000
Estimated ascending aortic growth rate*, mm/year	0.84 ± 0.41	0.93 ± 0.45	0.79 ± 0.37	0.000
Estimated aortic root growth rate*, mm/year	0.8 ± 0.16	0.86 ± 0.10	0.77 ± 0.18	0.000
AAD, *n*(%)	24 (1.52)	12 (2.32)	12 (1.13)	0.111

AAD, Stanford type A aortic dissection; GAM, generalized additive model; SD, standard deviation. Values are *n* (%), mean (SD). *Estimated growth rates of the ascending aorta and aortic root were calculated using a GAM.

### Follow-up and incidence of AAD

**Figure 2.**
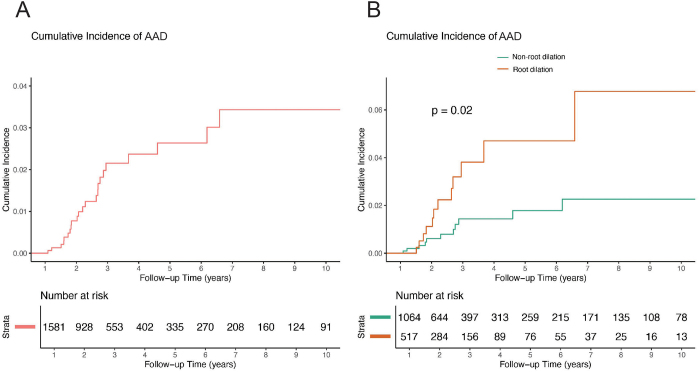
Cumulative incidence of AAD during follow-up. (A) Cumulative incidence curve of AAD for the overall cohort over a 10-year follow-up period. The number at risk for each group is displayed below the curve. (B) Cumulative incidence curves of AAD stratified by dilation type (root dilation: orange; non-root dilation: green). The comparison between groups was performed using the log-rank test, which showed a significantly higher incidence of AAD in patients with root dilation (*P* value = 0.02). The number at risk for each group is displayed below the curve. AAD, Stanford type A aortic dissection.

The median follow-up duration was 27.30 months (IQR: 18.98–49.42). AAD occurred in 24 patients during the follow-up period, with 12 cases in the root dilatation group (2.32%) and 12 cases in the non-root dilatation group (1.13%) (Table 3). The overall annual incidence of AAD was 0.43%. The annual incidence rate was higher in the root dilatation group (0.78%) compared to the non-root dilatation group (0.30%). Kaplan-Meier analysis revealed a 3-year cumulative incidence of 2.16% and a 5-year cumulative incidence of 2.64%, with a significant difference in the incidence of AAD between the groups (Fig. 2).

After univariate Cox regression and LASSO regression, aortic root index, hypertension, and root dilation were identified as risk factors for AAD and were included in the multivariable models (Supplementary Table 2, http://links.lww.com/JS9/E294 and Supplementary Figs 1–2, http://links.lww.com/JS9/E293). Following Firth-penalized Cox analysis, IPTW-weighted Cox analysis, and Fine–Gray analysis, all models consistently showed that patients with aortic root dilation had a more than two-fold increased risk of AAD compared to those without root dilation (Table 4).

**Table 4:** Multivariate analysis for Stanford type A aortic dissection

Variable	Firth-Penalized Cox Analysis			IPTW-Weighted Cox Analysis			Fine & Gray Analysis		
	HR	95%CI	*P* value	HR	95% CI	*P* value	SHR	95% CI	*P* value
Hypertension, *n*	3.49	1.55–7.86	0.004	3.50	1.43–8.59	0.005	3.55	1.59–7.51	0.002
Root dilation, *n*	2.62	1.17–5.86	0.019	2.81	1.18–6.73	0.016	2.28	1.04–5.03	0.040
Root diameter, mm	1.13	1.04–1.23	0.010	1.12	1.03–1.21	0.009	1.11	1.02–1.21	0.016

CI, confidence interval; HR, hazard ratio; IPTW, inverse probability of treatment weighting; SHR, subdistribution hazard ratio. SHR is estimated using the Fine and Gray competing risk model, where aortic surgery is treated as a competing event.

**Figure 3.**
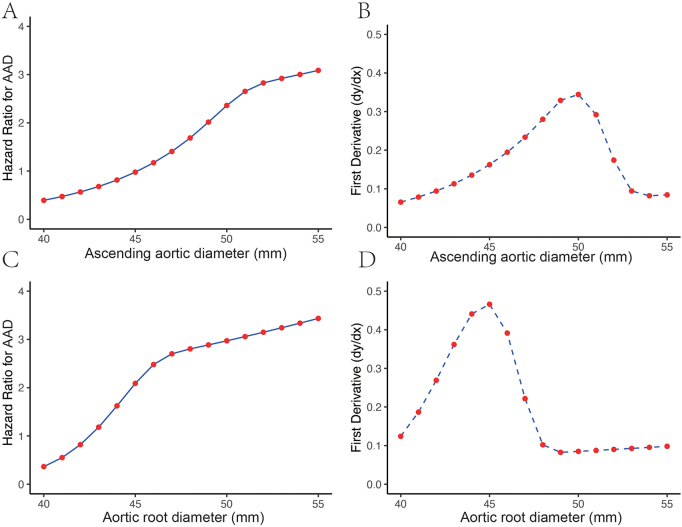
HR for AAD and the first derivative of HR with aortic diameter. (A) The relationship between the ascending aortic diameter (mm) and the HR for AAD. (B) The first derivative of the HR curve from panel (A), indicating the rate of change in HR (dHR/dx) as the ascending aortic diameter increases. (C) The relationship between the aortic root diameter (mm) and the HR for AAD. (D) The first derivative of the HR curve from panel (C), showing the rate of change in HR (dHR/dx) as the aortic root diameter increases. AAD, Stanford type A aortic dissection; HR, hazard ratio.

**Figure 4.**
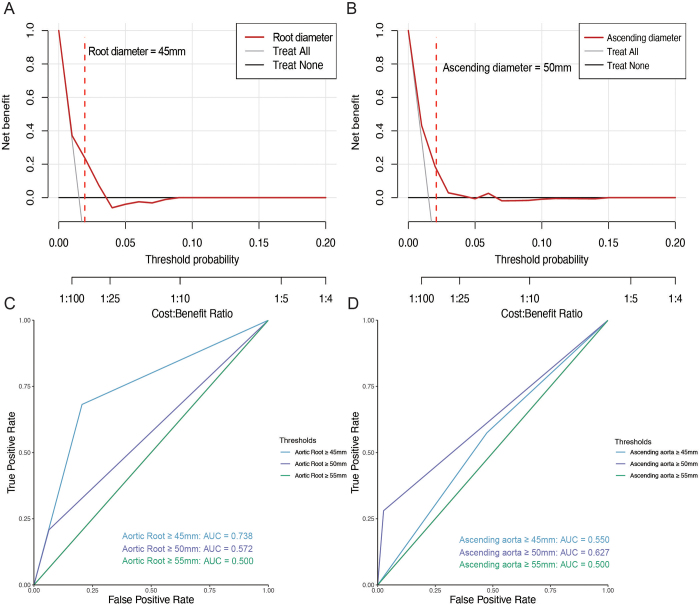
DCA and ROC curves for aortic dissection prediction based on aortic root and ascending aorta diameter thresholds. (A–B) DCA comparing the net clinical benefit of applying predefined surgical thresholds versus treating all or treating none. Panel A shows the DCA for an aortic root diameter ≥45 mm, and Panel B shows the DCA for an ascending aortic diameter ≥50 mm. Vertical dashed lines indicate the threshold probabilities corresponding to these diameter cutoffs. (C–D) ROC curves evaluating the discriminative performance of different aortic diameter thresholds for AAD. Panel C shows AUCs for aortic root diameter cutoffs of 45 mm (AUC = 0.738), 50 mm (AUC = 0.572), and 55 mm (AUC = 0.500). Panel D shows AUCs for ascending aortic diameter cutoffs of 45 mm (AUC = 0.550), 50 mm (AUC = 0.627), and 55 mm (AUC = 0.500). AAD, Stanford type A aortic dissection; AUC, area under the curve; DCA, decision curve analysis; ROC, receiver operating characteristic.

RCS analyses demonstrated that the hazard ratio (HR) for AAD increased with both baseline ascending aortic diameter and aortic root diameter. The rate of increase in HR peaked at an ascending aortic diameter of 50 mm, and the most rapid change in HR associated with aortic root diameter occurred at 45 mm. These findings suggest that 45 mm for the aortic root and 50 mm for the ascending aorta represent critical inflection points for AAD risk in BAV-related aortopathy (Fig. 3). DCA confirmed that the inflection points identified for aortic root diameter (45 mm) and ascending aortic diameter (50 mm) fell within the range of maximum net clinical benefit. Both inflection points showed superior net benefit over “treat-all” and “treat-none” strategies at low threshold probabilities, supporting their clinical utility as decision thresholds (Fig. 4A–B). Further ROC curve analysis validated the above findings, showing that the area under the curve (AUC) for predicting AAD was highest at a baseline aortic root diameter threshold of 45 mm and a baseline ascending aortic diameter threshold of 50 mm (Fig. 4C–D).

### Validation of guidelines

**Figure 5.**
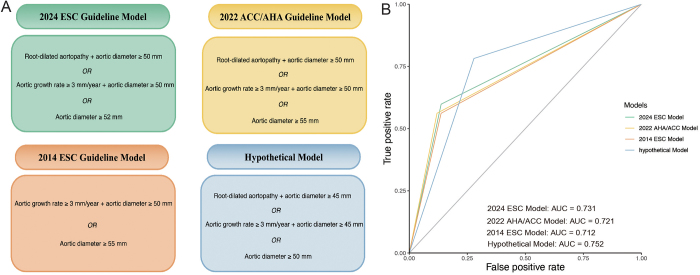
Assessment of guideline-based models for surgical timing: (A) Summary of surgical intervention thresholds for BAV-related aortic dilation in different guideline models and the hypothetical model. The figure summarizes the surgical intervention thresholds for BAV-related aortic dilation recommended by the 2014 ESC Guidelines, 2022 ACC/AHA Guidelines, 2024 ESC Guidelines, and the hypothetical model proposed in this study. (B) Comparative ROC curves for the four guideline-based models in predicting AAD risk. The hypothetical model proposed in this study achieves the highest predictive performance (AUC = 0.752), followed by the 2024 ESC model (AUC = 0.731), the 2022 ACC/AHA model (AUC = 0.721), and the 2014 ESC model (AUC = 0.712). AAD, Stanford type A aortic dissection; AUC, area under the curve; BAV, bicuspid aortic valve; ROC, receiver operating characteristic.

We constructed a regression model to simplify surgical indications based solely on aortic diameter and growth rate, using the 2014 ESC Guidelines, 2022 ACC/AHA Guidelines, and 2024 ESC Guidelines for BAV-related aortic disease. The 2024 ESC Guidelines model demonstrated superior predictive ability for AAD (AUC = 0.731) compared to the 2022 ACC/AHA model (AUC = 0.721) and the 2014 ESC model (AUC = 0.712). Adjusting the root diameter thresholds to 45 mm and the ascending aortic diameter thresholds to 50 mm further increased the predictive AUC to 0.752 (Fig. 5A-B).

### Surgical timing and AAD risk

**Figure 6.**
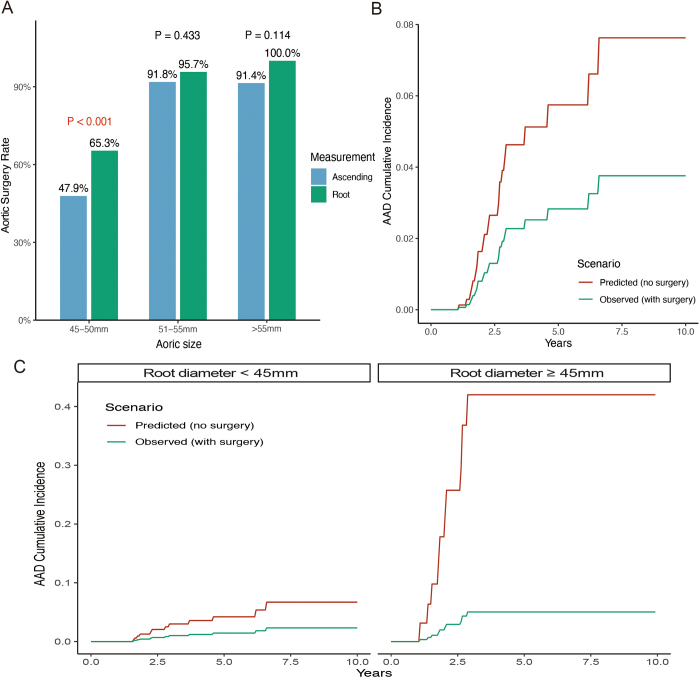
Surgical treatment patterns and competing risk analysis of AAD in patients with varying aortic diameters. (A) Aortic surgery rates stratified by aortic size (45–50, 51–55, and >55 mm) and measurement location (ascending aorta vs. aortic root). Surgery was more frequently performed for root diameters between 45 and 50 mm compared to ascending diameters in the same range (65.3% vs. 47.9%, *P* value < 0.001), whereas differences were not significant in larger size categories. (B) Cumulative incidence curves of AAD derived from a competing risk model using the Gray–Fine method. The predicted curve (red) represents the hypothetical cumulative incidence of AAD if all patients were assumed not to undergo surgery. The observed curve (green) shows the actual AAD incidence under real-world surgical practice. The divergence between the two curves highlights the potential protective effect of surgery on AAD occurrence. (C) Subgroup analysis of cumulative AAD incidence based on aortic root diameter: <45 mm (left) and ≥45 mm (right). Predicted incidence without surgery (red) was consistently higher than observed incidence with surgery (green), especially in patients with root diameters ≥45 mm, where the difference was more pronounced. Gray–Fine competing risk models were used in both subgroups, accounting for death as a competing event. AAD, Stanford type A aortic dissection.

Aortic surgery, treated as a competing event for AAD, was performed in 703 patients during the follow-up period of this study. The surgery rates across different aortic root and ascending aortic diameters are shown in Supplementary Figure 3, http://links.lww.com/JS9/E293. When the aortic root diameter was between 45 and 50 mm, the surgery rate was 65.3%, significantly higher than the surgery rate of 47.9% observed for ascending aortic diameters within the same range (*P* value < 0.001). However, when both the aortic root and ascending aorta exceeded 50 mm, the surgery rates showed no significant difference (Fig. 6A). These findings suggest that, in real-world clinical practice, surgical decision-making tends to be more aggressive in response to root dilation than to ascending aortic dilation.

We constructed Fine–Gray competing risk models to estimate the cumulative incidence of AAD under the current real-world surgical strategy of this study and to simulate the counterfactual risk assuming no surgical intervention in any patient. The results showed that the current surgical approach reduced the risk of AAD by 50.9%, 50.8%, and 50.7% at 3, 5, and 10 years, respectively (Fig. 6B). Among patients with an aortic root diameter <45 mm, the current strategy reduced AAD risk by 66.3%, 66.0%, and 65.3% at 3, 5, and 10 years, respectively. In patients with an aortic root diameter ≥45 mm, the corresponding risk reductions were consistently greater, with AAD risk lowered by approximately 88% at all three time points (Fig. 6C).

## Discussion

This study aimed to investigate the impact of different aortic dilation phenotypes on the risk of AAD in patients with BAV-associated aortopathy and to further evaluate more precise surgical intervention thresholds. The results showed that patients with aortic root dilation had a significantly higher incidence of AAD compared to those without root dilation. Aortic root index, root growth rate, and hypertension were identified as independent risk factors. Using Firth Cox, IPTW-weighted Cox, and Fine–Gray competing risk models, root dilation consistently emerged as a strong predictor of increased AAD risk, with a more than two-fold higher risk compared to non-root dilation. RCS analysis identified 45 mm for the aortic root and 50 mm for the ascending aorta as key inflection points for predicting AAD, which were further validated through ROC and DCA. Furthermore, we constructed a simplified prediction model based on two clinically relevant variables – diameter and growth rate – and found that the 2024 ESC guidelines demonstrated superior predictive accuracy in clinical settings. In addition, analysis of real-world surgical strategies revealed that patients with root dilation were more likely to undergo aortic surgery. These findings suggest that the current intervention thresholds for aortic dilation in BAV patients may warrant further reconsideration and potential refinement.

Previous studies have shown that patients with BAV are prone to ascending aortic dilation due to structural abnormalities of the aortic wall, such as poorly differentiated vascular smooth muscle cells and reduced expression of nuclear lamin A/C, which significantly increases the risk of AAD^[18,19]^. Michelena *et al* reported that BAV patients with an ascending aortic diameter ≥45 mm had an annual incidence of AAD of 0.45%, which is markedly higher than that of the general population[4]. Sundt *et al* observed an annual AAD incidence of approximately 0.1% among BAV patients with ascending aortic diameters ranging from 40 to 55 mm, with average diameters of 43.1 mm for the ascending aorta and 38.2 mm for the sinuses of Valsalva[20]. Our study’s overall AAD incidence of 0.43% aligns with Michelena’s results but includes patients with smaller aortic diameters. Compared to Sundt’s findings, our cohort exhibited a higher AAD rate, possibly due to demographic differences. Given that Asian populations typically have smaller BSA and BMI, the risk of AAD may be underestimated at equivalent aortic diameters.

BAV-associated aortopathy exhibits distinct patterns of dilation, which are internationally classified into root type, ascending type, and diffuse type[6]. Studies have shown that patients with root-type dilation are more likely to experience abnormal wall stress and progressive aortic expansion due to asymmetric valve leaflet structure and eccentric blood flow^[21,22]^. In cases of AAD, the aortic root is often the initial site of intimal tear, suggesting its central role in the disease pathogenesis^[23-25]^. De Feo *et al* also found that patients with the root phenotype exhibited faster aortic growth and a more aggressive disease progression[8].

Our study confirmed that patients with aortic root dilation exhibited significantly faster growth rates in both the aortic root and ascending aorta compared to those without root dilation, and their risk of AAD was more than twice as high. These findings underscore the significance of root dilation as a high-risk phenotype in BAV patients and highlight the need for earlier surgical intervention.

The ongoing changes in international guideline recommendations regarding surgical timing reflect the current controversy surrounding the optimal intervention threshold for BAV-related aortopathy. The 2014 ESC guidelines did not provide specific recommendations for root dilation[16]; the 2022 AHA/ACC guidelines subsequently recommended intervention at 50 mm for the root and 55 mm for the ascending aorta^[17]^. Most recently, the 2024 EACTS/STS and 2024 ESC guidelines lowered the intervention threshold for the ascending aorta to 52 mm^[9,10]^. This evolving trend illustrates a growing consensus toward earlier intervention, facilitated by advances in surgical techniques and reduced operative risk.

In our study, RCS analysis identified 45 mm for the aortic root and 50 mm for the ascending aorta as key inflection points for a marked increase in AAD risk. These thresholds were further validated by ROC and DCA, demonstrating superior predictive performance. Compared to the current recommendations from the 2022 AHA/ACC (50 mm for the root, 55 mm for the ascending aorta), 2024 ESC, and 2024 EACTS/STS (50 mm for the root, 52 mm for the ascending aorta) guidelines, our proposed thresholds offer improved risk stratification for identifying high-risk patients. Wu *et al* also supported the use of 50 mm as a more appropriate surgical threshold in a cohort of 964 patients with ascending aortic aneurysms, further validating our findings[26].

In this study, a total of 703 patients underwent aortic surgery, including ascending aortic replacement and root replacement. We found that, in real-world clinical practice, surgical thresholds appear to be more aggressive for aortic root dilation than for isolated ascending aortic dilation. Specifically, 65.3% of patients with aortic root diameters between 45 and 50 mm underwent surgery, compared to only 47.9% of those with ascending aortic diameters in the same range. Furthermore, competing risk modeling demonstrated that the current surgical strategy reduced the risk of AAD by approximately 88% in patients with aortic root diameters ≥45 mm. These findings support the central hypothesis of this study – that aortic root dilation in BAV-associated aortopathy represents a more unstable phenotype that may warrant earlier surgical intervention than currently recommended in existing guidelines.

In determining the timing of surgery, it is also essential to consider procedural risk and long-term outcomes. For BAV patients, root replacement is commonly performed using either the Bentall procedure or valve-sparing root replacement (VSRR). Previous studies have shown that both techniques are associated with low perioperative risk and excellent long-term outcomes, with 5-year survival and freedom-from-reoperation rates exceeding 95%[27]. More recent evidence further supports that individualized surgical approaches using Bentall or VSRR in BAV patients with root aneurysms can achieve outstanding long-term clinical and functional results[28]. Taken together, the low surgical risk and favorable long-term outcomes associated with root intervention suggest that earlier surgical treatment may be appropriate for BAV patients with root dilation, particularly when the diameter exceeds 45 mm.

## Limitations

This study has several limitations. First, it was a single-center, retrospective analysis, which may limit the external validity of our findings. Although the sample size was large, future multicenter, prospective studies are needed to validate these results. Second, the number of AAD events was limited; although we applied Firth’s penalized Cox regression and conducted validation using real-world surgical data and counterfactual modeling, the risk of model overfitting due to sparse events cannot be entirely ruled out. Third, the imaging data were derived from 2D echocardiography, which, while widely used, has limited measurement precision. Although quality control was ensured, future studies should incorporate advanced imaging modalities (e.g., CT, MRI) and circulating biomarkers to improve accuracy and risk prediction.

## Conclusion

This study highlights the distinct phenotypic differences in BAV-related aortopathy and emphasizes the elevated risk of AAD in root-dilated patients. Early surgical intervention should be considered for root-dilated BAV patients. The 2024 ESC guidelines provide more accurate thresholds for surgical intervention and demonstrate improved predictive performance in preventing AAD. However, further research is needed to refine surgical decision-making in BAV-related aortopathy, considering patient-specific factors.

## Data Availability

Data will be made available from the corresponding author upon reasonable request.

## Research registration unique identifying number (UIN)

Name of the registry: Research Registry.

Unique identifying number or registration ID: researchregistry10863.
